# Functional and Topological Conditions for Explosive Synchronization Develop in Human Brain Networks with the Onset of Anesthetic-Induced Unconsciousness

**DOI:** 10.3389/fncom.2016.00001

**Published:** 2016-01-21

**Authors:** Minkyung Kim, George A. Mashour, Stefanie-Blain Moraes, Giancarlo Vanini, Vijay Tarnal, Ellen Janke, Anthony G. Hudetz, Uncheol Lee

**Affiliations:** ^1^Department of Anesthesiology, University of Michigan Medical SchoolAnn Arbor, MI, USA; ^2^Center for Consciousness Science, University of Michigan Medical SchoolAnn Arbor, MI, USA; ^3^Department of Physics, Pohang University of Science and TechnologyPohang, South Korea; ^4^Neuroscience Graduate Program, University of Michigan Medical SchoolAnn Arbor, MI, USA

**Keywords:** explosive synchronization, state transition, anesthesia, brain network, consciousness

## Abstract

Sleep, anesthesia, and coma share a number of neural features but the recovery profiles are radically different. To understand the mechanisms of reversibility of unconsciousness at the network level, we studied the conditions for gradual and abrupt transitions in conscious and anesthetized states. We hypothesized that the conditions for explosive synchronization (ES) in human brain networks would be present in the anesthetized brain just over the threshold of unconsciousness. To test this hypothesis, functional brain networks were constructed from multi-channel electroencephalogram (EEG) recordings in seven healthy subjects across conscious, unconscious, and recovery states. We analyzed four variables that are involved in facilitating ES in generic, non-biological networks: (1) correlation between node degree and frequency, (2) disassortativity (i.e., the tendency of highly-connected nodes to link with less-connected nodes, or vice versa), (3) frequency difference of coupled nodes, and (4) an inequality relationship between local and global network properties, which is referred to as the suppressive rule. We observed that the four network conditions for ES were satisfied in the unconscious state. Conditions for ES in the human brain suggest a potential mechanism for rapid recovery from the lightly-anesthetized state. This study demonstrates for the first time that the network conditions for ES, formerly shown in generic networks only, are present in empirically-derived functional brain networks. Further investigations with deep anesthesia, sleep, and coma could provide insight into the underlying causes of variability in recovery profiles of these unconscious states.

## Introduction

The mechanisms of the emergence from unconsciousness in sleep, anesthesia, and coma are still elusive. Consistent network features of the unconscious state have been reported, including the reconfiguration of functional brain networks with inhibited long range connections, reduced information transmission, disrupted hub structures, and increased functional modularity (Alkire et al., 2008; Boveroux et al., 2010; Ferrarelli et al., 2010; Ku et al., 2011; Schröter et al., 2012; Jordan et al., 2013; Lee et al., 2013a,b, 2015; Barttfeld et al., 2015). Diverse anesthetics reduce network communication and the capacity of information integration (Lee et al., 2009, 2013a; Casali et al., 2013), which is thought to be necessary for consciousness (Mashour, 2013; Oizumi et al., 2014). However, how an inefficient, disrupted brain network is able to rapidly return to a normal configuration and support conscious brain function remains to be answered. In this study, we hypothesized that, despite the anesthetic-mediated disruption of information integration, brain networks just past the threshold for the loss of consciousness have network conditions that can allow rapid recovery.

A series of studies since 2011 investigated the mechanisms of explosive synchronization (ES) in complex networks (Gómez-Gardeñes et al., 2011; Leyva et al., 2013; Li et al., 2013; Liu et al., 2013; Skardal et al., 2013; Zhang et al., 2013, 2014, 2015; Zhu et al., 2013; Skardal and Arenas, 2014; Navas et al., 2015; Sendiña-Nadal et al., 2015). ES was originally described as an abrupt transition from incoherent to synchronized states at a critical coupling strength in a model network. In a general synchronization path, pairs of nodes are entrained to a more extensive pattern of synchronization, and the likelihood of two synchronized clusters merging is proportional to their size. Once a large cluster has formed, it dominates the system, absorbing any smaller clusters that merge and grow locally. However, with ES, each cluster of synchronization grows, but the growth of the largest cluster is suppressed. This allows many large but disconnected clusters to grow, until the system reaches the critical threshold where a small perturbation of connection or synchronization strength triggers a rapid transition to global synchronization. All clusters combine at once in a single explosive unification. Zhang et al. (2014) explained the mechanism of this abrupt synchronization path by introducing the “suppressive rule” (i.e., suppression of the largest cluster driving a gradual synchronization process) as a necessary condition for synchronization between pairs of nodes. This process is similar to explosive percolation (Zhang et al., 2014; D'souza and Nagler, 2015), where it has been shown that ES in dynamical phase space occurs by the same mechanism as the explosive percolation in configuration space.

Various network conditions for ES have been identified. ES can occur with small network perturbations if: (1) the natural frequencies of oscillators and the degrees of nodes in a scale-free network have a positive correlation (Gómez-Gardeñes et al., 2011; Liu et al., 2013); (2) a network has reduced degree disassortativity (i.e., the tendency of highly-connected nodes to link with less-connected nodes, or vice versa) in various network types (Li et al., 2013; Liu et al., 2013; Zhu et al., 2013; Sendiña-Nadal et al., 2015); (3) a network has large frequency differences in connected oscillators (Leyva et al., 2013; Zhu et al., 2013; Skardal and Arenas, 2014); and (4) the frequency difference of coupled nodes is larger than a critical value that is determined by the global network properties (the product of global coupling strength and phase synchronization) according to the suppressive rule (Zhang et al., 2014, 2015). In general, an ES transition is associated with the phenomenon of hysteresis, which has been observed in the brain during anesthetic state transitions (Kelz et al., 2008; Friedman et al., 2010; Joiner et al., 2013).

In this study, we investigated whether the four known network conditions for ES also hold in a functional brain network derived from multichannel EEG from seven healthy human volunteers. The synchronization of large populations of neurons is thought to be a necessary condition for the conscious state (Laureys and Tononi, 2011). Accordingly, we assumed that the breakdown of temporal coordination through synchrony in brain networks would reflect the state transition between conscious and unconscious states. We used the inhaled anesthetic sevoflurane to gradually modulate the level of consciousness across multiple states: eyes-closed waking, unconsciousness, recovery, and the transitions between. Our empirical findings using graph-theoretical network analysis from human EEG directly supported the hypothesis, demonstrating that slow titration of the inhaled anesthetic sevoflurane results in a dose-dependent reconfiguration of network topology and dynamics, including: (1) increased positive correlation between node degree and frequency, (2) diminished degree disassortativity and disruption of strong hub structures found in the resting state, (3) increased frequency difference between coupled nodes, and (4) absence of a modified definition of the suppressive rule. All of these properties are known to impair normal network synchronization and create conditions for ES, which can occur with small network perturbations.

## Methods

### Anesthesia and EEG recording

This study was conducted at the University of Michigan Medical School and was approved by the Institutional Board Review (HUM00061087). Seven healthy volunteers (4 males, 20–23 years of age) who gave their written informed consent participated in this study. Participants who were pregnant, or with a history of obstructive sleep apnea, gastroesophageal reflux, cardiac conduction abnormalities, asthma, epilepsy, history of problems with anesthesia, family history of problems with anesthesia, history of drug use, and any neurologic or psychiatric history were excluded from this study. Participants kept their eyes closed during waking states throughout the experiment and were administered sevoflurane anesthesia by mask inhalation with a tight seal at an initial concentration of 0.4% in high-flow oxygen (8 L/min). Sevoflurane was allowed to equilibrate for 15 min at each specified concentration. After equilibration, there was a 10 min recording period at the target concentration. EEG data for a given concentration reflect steady-state levels only, after equilibration occurred. Sevoflurane concentration was increased by levels of 0.2% until loss of consciousness (LOC) was achieved, as judged by the surrogate of loss of behavioral responsiveness. After a 10-min period of LOC, we performed the reverse protocol until participants recovered consciousness. After recovery of consciousness (ROC), anesthetic concentration was titrated downward until end-tidal values were 0%. Responsiveness was assessed every 30 s by compliance with auditory instruction to squeeze an object in the hand (randomized to right or left). EEG data were acquired using a 64-channel sensor net from Electrical Geodesics, Inc. (Eugene, OR) with a sampling frequency of 500 Hz. All channels were referenced to the vertex with impedance reduced to below 50 KΩ before data collection. After the EEG data were collected, signals were high-pass filtered at 0.1 Hz, and re-referenced to an average reference. An investigator visually inspected the data and removed channels or epochs with noise and artifacts (Blain-Moraes et al., 2015).

### State of consciousness

For each participant, we identified two key time points in the experiment; (1) LOC: The first non-response to an auditory command during induction followed by at least 5 min of 0% responsiveness; and (2) ROC: The first response to an auditory command after LOC. Based on these two time points, we defined the following five 5-min states: (1) Baseline: Before sevoflurane administration, eye-closed resting state (100% responsiveness); (2) Transition to unconsciousness (Trans_UN_): During induction, immediately before LOC (>0% responsiveness); (3) Unconscious (UCS): Between LOC and ROC (0% responsiveness); (4) Transition to recovery (Trans_CON_): During emergence, immediately after ROC (>0% responsiveness); (5) Recovery: After emergence, eye-closed resting state (100% responsiveness; Blain-Moraes et al., 2015).

### EEG network analysis

We divided the signal into several frequency bands including delta (1–4 Hz), theta (4–8 Hz), alpha (8–13 Hz), beta (13–30 Hz), and broadband (1–50 Hz) using band-pass filtering methods. In this paper, we focused on the alpha band because of its characteristic connectivity and topography directly reflecting various states of consciousness induced by anesthesia (Cimenser et al., 2011; Lee et al., 2013b; Purdon et al., 2013). No significant results were observed in the other bands; results from other bandwidths are presented in the Supplementary Figures.

To construct a functional brain network from EEG, we used the weighted phase lag index (WPLI) (Vinck et al., 2011), which is a robust method that reduces the volume conduction problem.

(1) $$WPLI_{ij}=\frac{\left|E\{ℑ(C_{ij})\}\right|}{E\{\left|ℑ(C_{ij})\right|\}}=\frac{\left|E\left\{\left|ℑ(C_{ij})\right|sgn(ℑ(C_{ij}))\right\}\right|}{E\{\left|ℑ(C_{ij})\right|\}}$$

where ℑ(*C*_*ij*_) is an imaginary part of cross-spectrum *C*_*ij*_ between two signals *i* and *j*. The cross-spectrum *C*_*ij*_ is defined as $Z_{i}Z_{j}^{*}$, where *Z*_*i*_ is the complex value Fourier spectra of the signal *i* for each frequency, and $Z_{j}^{*}$ is the complex conjugate of *Z*_*j*_. *C*_*ij*_ can be written as *Re*^*iθ*^, where *R* is magnitude and θ is the relative phase between signal *i* and *j*. If the phases of one signal (*i*) always lead or lag those of the other signal (*j*), that is, that is, *Pr*{*sgn*(ℑ(*C*_*ij*_)) = 1 *or* − 1}, then *WPLI*_*ij*_ equals 1. On the other hand, if the phase lead/lag relationship of two signals is random, the *WPLI*_*ij*_ value is 0.

Based on the WPLI matrix, we constructed a binary adjacency matrix *A*_*ij*_. If the *WPLI*_*ij*_ value of nodes *i* and *j* is larger than a threshold, the connection is equal to 1, otherwise, it equals 0. To find an threshold for the given data, we tested the effect of varying thresholds (ranging from the top 30% to the 70% of WPLI values) on the network properties. All thresholds showed similar results, thus, in this study we used the binary networks of the top 30% of WPLI for further analysis.

We calculated basic network topological properties such as node degree, betweenness centrality (BC), global efficiency (GE) (reflecting information integration capacity) and modularity (reflecting local functional segregation). The degree is defined as the number of connections for each node. The BC is a measure of the influence of a node on the information transmission in a network through the facilitation of shortcuts. It is calculated by the fraction of the shortest paths passing through a node with respect to all possible shortest paths in a network. The GE is evaluated by an inverse of the average shortest path length over all pairs of nodes; the shorter the path length, the higher the efficiency. The modularity was calculated using the Louvain algorithm with a brain connectivity toolbox (Rubinov and Sporns, 2010). The node degree and BC were used to define hubs in a network. The GE and modularity were used to estimate the global functional integration and local functional segregation of a network, respectively.

The assortativity of a network is the degree to which nodes have a preference to attach to other nodes that have similar node degree. Previous studies revealed that the assortativity plays an important role for gradual/abrupt synchronization in a network of Kuramoto oscillators (Li et al., 2013; Liu et al., 2013; Zhu et al., 2013; Sendiña-Nadal et al., 2015). The simulation demonstrated that highly positive or negative assortativity induces a gradual state transition, while a small or neutral assortativity facilitates a sudden state transition in a network. We computed the assortativity by Newman's algorithm (Newman, 2002),

(2) $$a=\;\frac{L^{-1}\sum_{i}j_{i}k_{i}-{\left[L^{-1}\sum_{i}\frac{1}{2}(j_{i}+k_{i})\right]}^{2}}{L^{-1}\sum_{i}\frac{1}{2}(j_{i}^{2}+k_{i}^{2})-{\left[L^{-1}\sum_{i}\frac{1}{2}(j_{i}+k_{i})\right]}^{2}}$$

where *j*_*i*_ and *k*_*i*_ are the degrees of the nodes at the ends of the *i*_*th*_ link, with *i* = 1, …*L*. The assortativity is bounded within the range −1 ≤ *a* ≤ 1. If *a* is positive (or negative), the network has an assortative (or disassortative) feature. Thus, if a network is disassortative, it indicates that high-degree nodes are more likely connect with the small-degree nodes, and vice versa. If *a*~0, the network does not have such a bias.

The suppressive rule, introduced by Zhang et al. (2014), is a necessary condition for synchronization of coupled nodes in a Kuramoto network with a positive correlation between node degrees and coupling strengths (see Table 1 for the details). It is applicable regardless of the type of network structure. The suppressive rule is presented as an inequality relationship between frequency difference of coupled nodes and network properties (local order parameters and coupling strength) in a network. According to the suppressive rule in the Kuramoto network, if the frequency difference of coupled nodes is larger than a certain threshold, the synchronization of the coupled nodes are suppressed. On the other hand, if the frequency difference is smaller than the threshold, the two nodes are synchronizable. In this experiment, EEG was collected over varying states of consciousness, resulting in time-varying global network properties (order parameter and coupling strength), and frequency differences of coupled nodes. In order to investigate the relationship between the brain network and behavioral state, we examined the frequency difference, global network properties, and their inequality relationship over time during the whole experiment.

**Table 1:** **Glossary of terms**.

**Keywords**	**Descriptions**
First-order phase transition	Discrete changes from incoherent to synchronized state or vice versa, as the coupling strength of coupled oscillators increases or decreases, respectively. A more continuous change is referred to as a “second order phase transition.”
Explosive synchronization (ES)	A phenomenon characterized by first-order phase transition between incoherent and synchronized states in a network of coupled oscillators.
Kuramoto model	Mathematical model to study collective behavior of large scale coupled phase oscillators in physical and biological systems. The model consists of N phase oscillators, their natural frequencies (ω_*i*_), coupling strengths (σ), coupling structure (*a*_*ij*_), and phase differences of coupled oscillators (θ_*j*_ − θ_*i*_) as following, ${\dot{\theta }}_{i}=\omega_{i}+\sigma \sum_{j=1}^{N}a_{ij}\sin(\theta_{j}-\theta_{i})$
Suppressive rule	A necessary condition for synchronization of two coupled oscillators. The condition was analytically derived for coupled Kuramoto oscillators with a positive correlation between node degrees and coupling strengths (σ → σ|ω_*i*_|∕*k_i_*). Here, *k*_*i*_ is the node degree.
Order parameter	A measure for average phase coherence of the population of oscillators.
Weighted phase lag index (WPLI)	A measure of phase locking based on phase lead and lag relationship, which helps reduce (but cannot eliminate) the volume conduction problem of EEG signal.
Node degree	The number of edges/links connected to a node in a network.
Betweenness centrality (BC)	A measure of the extent to which a node acts as a bridge that creates the shortest path between two other nodes.
Hub	A node with higher node degree or larger betweenness centrality. Hubs play an important role in information integration in a network. Anesthesia reduces or redistributes hub strength (node degree and BC) in brain networks.
Modularity	A measure reflecting the strength of division of a network into functional units. Anesthesia increases the modularity of brain networks.
Global efficiency (GE)	The inverse of average shortest path lengths over all pairs of nodes. This measure reflects the capacity of global integration of a network. Anesthesia reduces global efficiency.
Disassortativity	A “preference” for higher degree nodes to connect with lower degree nodes, or vice versa. Anesthesia diminishes the preference of the brain network, thereby randomizing the connectivity.

First, we determined the peak frequency of the EEG signal (i.e., the frequency with the maximal power in the spectrum) within the alpha frequency range (8–13 Hz). We segmented the EEG into 10-s epochs, and applied the power spectrum density function (“psd.m” in Matlab, with 5 s Hanning windows and 1 s overlaps). The average peak frequency over all epochs within the states was defined as an average of the peak frequency of the EEG signals. A frequency difference *Y*_*ij*_ between two coupled nodes (i.e., EEG channels) in the network was determined by the difference between two peak frequencies as follows.

(3) $$Y_{ij}=\frac{\left|f_{i}-f_{j}\right|}{\left|f_{i}|+|f_{j}\right|}$$

The *f*_*i*_ is the peak frequency of node *i* for a frequency band, and *Y*_*ij*_ is a normalized frequency frequency difference between two nodes *i* and *j*. *Y*_*ij*_ will be 0 if *f*_*i*_ = *f*_*j*_, otherwise 0 < *Y*_*ij*_ < 1. The global frequency difference for a network is defined with the average *Y*_*ij*_ for all connected nodes.

We considered local synchronization conditions based on the extended local suppressive rule, which was introduced for a network of Kuramoto oscillators (Zhang et al., 2014; Navas et al., 2015). The local suppressive rule for each pair of coupled nodes is defined as the following inequality relationship,

(4) $$Y_{ij}\leq \lambda (r_{i}+r_{j})$$

where λ is the absolute coupling strength and the *r*_*i*_ is a local order parameter of *i*_*th*_ Kuramoto phase oscillator. To apply this local suppressive rule to brain networks, we replaced the coupling strength λ* with* 1−*c*, where *c* is the rescaled anesthetic concentration (from 0 to 1). We assumed that the long-range coupling strength of neural populations is inversely correlated with anesthetic concentration (Moon et al., 2015). Furthermore, the local order parameter *r*_*i*_ of a node *i* in Equation (4) was estimated as the averaged *WPLI* over the connected nodes, $r_{i}=\frac{1}{N}\;\overset{N}{\underset{j}{\sum }}A_{ij}WPLI_{ij}$, where *N* is the degree of node *i*. In the model, *Y*_*ij*_ is the difference of natural frequencies of Kuramoto However, for the application to EEG, we assumed that the observed frequencies in a time window would serve as the initial frequencies in the very next time window in a non-stationary and dynamic brain. Thus, if the variability of the observed frequencies for a state is small enough to differentiate the conscious and unconscious states, we consider the observed frequencies are similar to the natural frequencies in the model for a state.

To account for a suppressive anesthetic effect on local synchronization, we modified the local suppressive rule for all coupled nodes. The status of coupled nodes *i* and *j*, *e*_*ij*_, was defined as suppressive or non-suppressive as follows:

(5) $$s_{ij}=Y_{ij}-\lambda (r_{i}+r_{j})$$

(6) $$e_{ij}=\{\begin{array}{cc} 1,if\;s_{ij}>0, & Suppressive\\ 0,if\;s_{ij}\leq 0, & Non–suppressive \end{array},$$

where λ = 1 − *c and c* is anesthetic concentration scaling 0 as a minimum dose (baseline conscious state) and 1 as a maximum dose (unconscious state). In the “suppressive” state, the synchronization of coupled nodes is inhibited, whereas two nodes are synchronizable in the “non-suppressive” state.

The local suppression strength (*S*_*i*_) of a node was calculated by counting the status of all connected nodes:

(7) $$S_{i}=\;\frac{1}{L_{i}}\sum_{j}^{L_{i}}e_{ij}$$

where *L*_*i*_ is the degree of *i*_*th*_ node.

Furthermore, we calculated the regional suppression strengths (*S*_*r*_) in the brain network (prefrontal, frontal, central, temporal, parietal, and occipital regions according to the EEG channel configuration) and measured which brain region has the strongest suppression strength during anesthesia. The global suppression strength *S* was obtained by taking average of the all *S*_*i*_.

**Figure 1:**
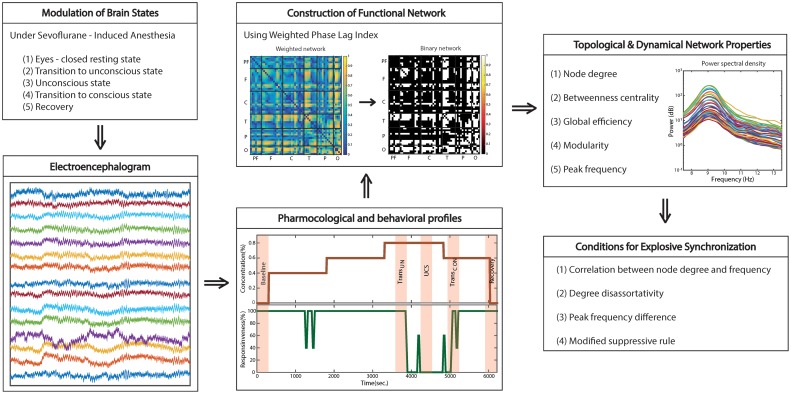
**Schematic diagram of study design**. The functional brain network topology and dynamics were analyzed with high-density EEG recorded across states: eye-closed resting; transition to unconsciousness; unconsciousness; transition to consciousness; recovery. To construct a functional network, we used weighted phase lag index (WPLI), which is relatively robust to the volume conduction problem of EEG. Four network conditions for explosive synchronization (ES) reported in generic networks were applied to the brain network and investigated for each state, as well as typical properties of network topology and dynamics.

The whole study design and analysis are illustrated in Figure 1.

### Statistical analysis

We performed the Kruskal-Wallis test (“kruskalwallis.m,” Matlab statistical toolbox), which is a nonparameteric version of a classical one-way ANOVA with Tukey's multiple comparisons (“multcompare.m” with alpha = 0.05 and ctype = “tukey-kramer”) for node degree, BC, and peak frequency, taking the average of EEG channels in the anterior (prefrontal and frontal) and posterior (occipital) brain regions of each subject. We also performed a repeated-measures One-way ANOVA and a post hoc analysis with Tukey's multi-comparison test for GE, modularity, disassortativity, and frequency difference comparisons across the states, treating each participant as an independent sample. For the correlation between node degree and frequency, we also treated each subject as one sample taking the average of node degree and peak frequency respectively for all epochs across the states. Significance was obtained by using exact permutation distributions with the null hypothesis of no correlation against the alternative that the result had nonzero correlation. The statistical significance between global S with change of concentration and responsiveness was also performed using repeated One-way ANOVA with Tukey multiple comparison. MATLAB (Natick, MA) was used for the statistical tests and differences were considered significant at adjusted *P*-values less than 0.05 (for the figures, ^*^P < 0.05, ^**^P < 0.01, and ^***^P < 0.001).

## Results

### Reconfiguration of brain network structure and dynamics across states of consciousness

**Figure 2:**
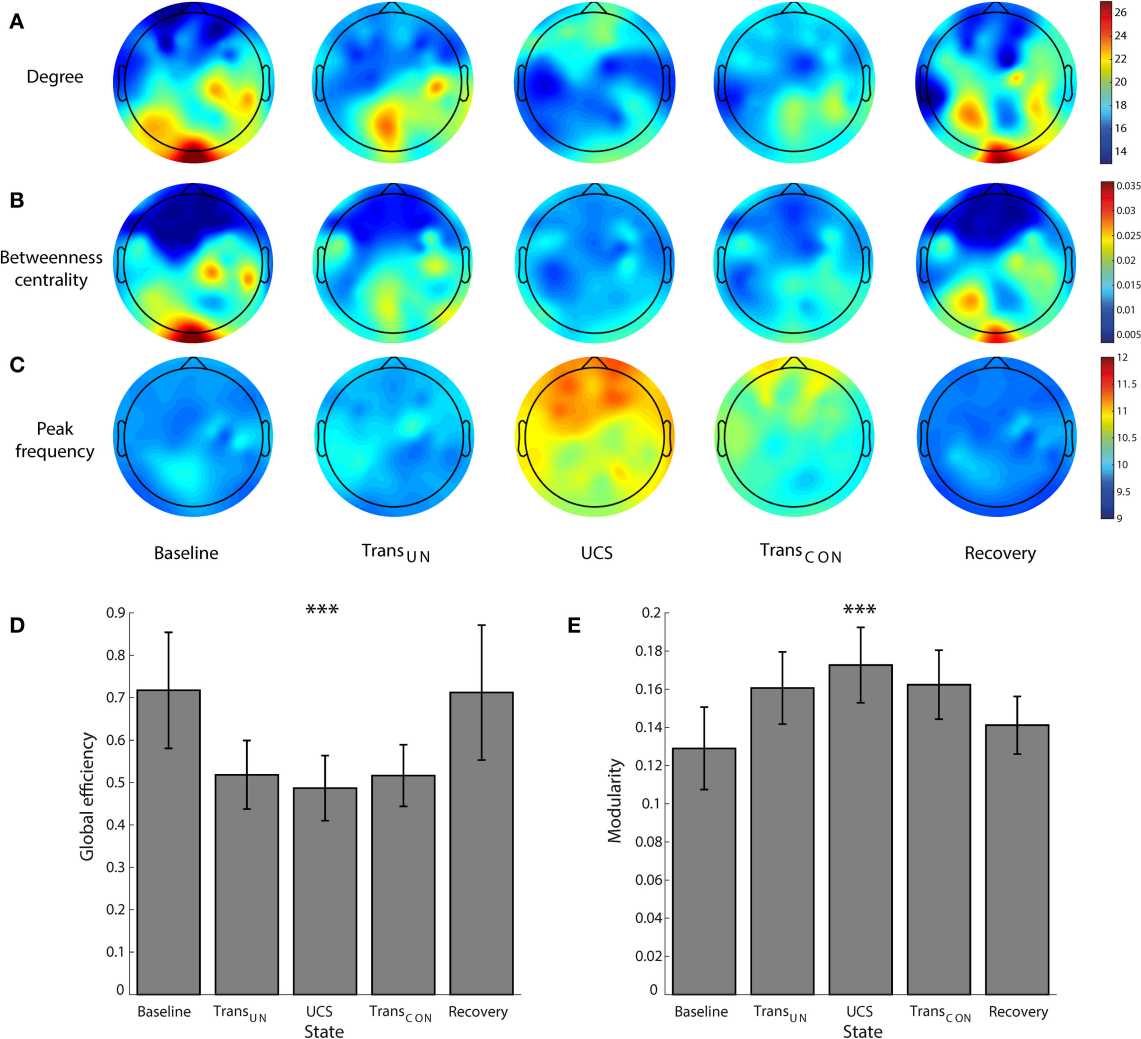
**The reconfiguration of brain network structure and dynamics during sevoflurane anesthesia**. For the alpha band, the changes of **(A)** the topography of node degree, **(B)** betweenness centrality, **(C)** peak frequency (Hz), **(D)** global efficiency, and **(E)** modularity are presented across five states: baseline, transition to unconsciousness (Trans_*UN*_), unconscious state (UCS), transition to consciousness (Trans_*CON*_), and recovery. The disruption and recovery of posterior hub structures, as defined by higher node degree and betweenness centrality, is obvious with loss and recovery of consciousness (node degree, *p* < 0.05; BC, *p* < 0.01). An increase of peak frequency in the unconscious state is also observed (*p* < 0.05). In addition, the reduced global efficiency and increased modularity in UCS are significant. Error bars indicate standard deviation for seven subjects. Significance level using ANOVA: ^***^p < 0.001.

Figure 2 demonstrates the overall changes across states of brain network structure (node degree and BC) and dynamics (peak frequency) as well as global efficiency (GE) and modularity. In the resting state with eyes closed, the occipital region has a higher node degree and BC, i.e., it is the primary hub structure (Figure 2B). However, anesthesia significantly inhibits the posterior hubs in the unconscious state (node degree, *p* < 0.05; BC, *p* < 0.01), which recovers along with consciousness (BC, *p* < 0.01). Figure 2C demonstrates that after induction of unconsciousness, the peak frequency of alpha power distribution is increased, and shifted to the frontal region in the unconscious state (Baseline vs. Trans_*UN*_, *p* < 0.05; Baseline vs. UCS, *p* < 0.05; UCS vs. Recovery, *p* < 0.05). Notably, separate analysis on the same data demonstrated that the total power of alpha band (over 8–13 Hz) did not consistently increase and there was no consistent anteriorization during unconsciousness (Blain-Moraes et al., 2015). We also observed disrupted GE and increased modularity during unconsciousness, as shown in Figures 2D,E (GE: Baseline vs. Trans_*UN*_, *p* < 0.001; Baseline vs. UCS, *p* < 0.01; Baseline vs. Trans_*CON*_, *p* < 0.05; Trans_*UN*_ vs. Recovery, *p* < 0.05; UCS vs. Recovery: *p* < 0.001; Trans_*CON*_ vs. Recovery, *p* < 0.05, Modularity: *p* < 0.001; Baseline vs. Trans_*UN*_, *p* < 0.05; Baseline vs. UCS, *p* < 0.01; UCS vs. Recovery, *p* < 0.05). These characteristic changes of network structures and dynamics were only observed in the alpha band (see Figures S1–S3 for the other frequency bands).

### Change of correlation between node degree and peak frequency across states

**Figure 3:**
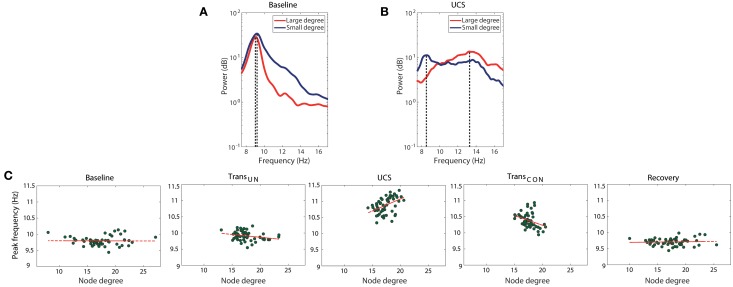
**Change of the correlation between node degree and frequency across states**. The peak frequencies of alpha power for high (red line) and low (blue line) degree nodes are compared in **(A)** baseline and **(B)** UCS for a subject as an example. The high degree node has a larger peak frequency in UCS, whereas there is no significant dependency of peak frequency on node degree in baseline. The range of peak frequencies is also wider in UCS. **(C)** The correlation between average node degree and average peak frequency changes significantly across states. The node degree and peak frequency for a node is expressed as average values over seven subjects. There is a positive correlation between degree and peak frequency in UCS (Spearman coefficient of 0.473, *p* < 0.001), while no correlation appears in baseline (Spearman coefficient of −0.024, *p* = 0.86) and recovery (Spearman coefficient of −0.022, *p* = 0.87) states of consciousness. However, the transitional states showed relatively small negative correlations. The results suggest that the brain network in the UCS is in a condition primed for abrupt state transition, whereas the other states are in a condition conducive to gradual state transition.

The correlation between node degree and frequency in a network is one condition for ES. It has been demonstrated in a previous study with generic networks that positive and negative correlations between node degree and frequency produce abrupt and gradual synchronizations, respectively (Liu et al., 2013). In brain networks, the abrupt and gradual synchronizations could be associated with fast and slow transitions between unconscious and conscious states. Figures 3A,B show the relationship between peak frequency and node degree in an EEG network for the alpha band, and how the relationship changes across states. To determine the peak frequency, we chose two EEG channels from prefrontal and central regions, and measured their peak frequencies and node degrees in the EEG network. The averaged peak frequency and node degree were calculated from segmented 10 s epochs for each EEG channel. It can be seen in one participant that during UCS (Figure 3B), the higher degree node (21.33) has a higher peak frequency (13.3 Hz) and the lower degree node (15.46) has a lower peak frequency (8.5 Hz). However, in baseline (Figure 3A), the same two EEG channels (with average node degree of 15.42 and 10.54, respectively) did not show any significant difference in terms of peak frequency. We observed that the average power of the alpha band is slightly decreased, and the peak frequencies are shifted to a comparatively higher frequency range in UCS. In addition, the range of peak frequency distribution (dotted lines in Figures 3A,B) becomes broader in UCS than baseline.

The change of correlation between node degree and peak frequency across states is clear. Figure 3C presents the relationship between the average node degrees and the average peak frequencies for all seven subjects. The node degree and peak frequency for a specific EEG channel were obtained by averaging over seven subjects. In Figure 3C, the average peak frequencies of nodes in baseline are narrowly distributed while the average node degrees are broadly distributed. The baseline did not show any correlation (Spearman coefficient of −0.024, *p* = 0.86). However, the two transitional states presented weak negative correlations (Trans_*UN*_: Spearman coefficient of −0.282, *p* < 0.05, Trans_*CON*_: Spearman coefficient of −0.343, *p* < 0.01). A significant positive correlation was observed in UCS (Spearman coefficient of 0.473, *p* < 0.001), suggesting that the brain network during a lightly anesthetized state is predisposed to an abrupt state transition, whereas the other states are predisposed to gradual transition. This is the first empirical observation of a significant linear correlation between the peak alpha frequency and node degree in the brain. After the recovery of consciousness, the correlation diminished (Spearman coefficient of −0.022, *p* = 0.87). The correlations between node degree and frequency for all individual subjects and states are presented in Figure S6.

### Change of topological and dynamical network conditions for explosive synchronization (ES) across states

**Figure 4:**
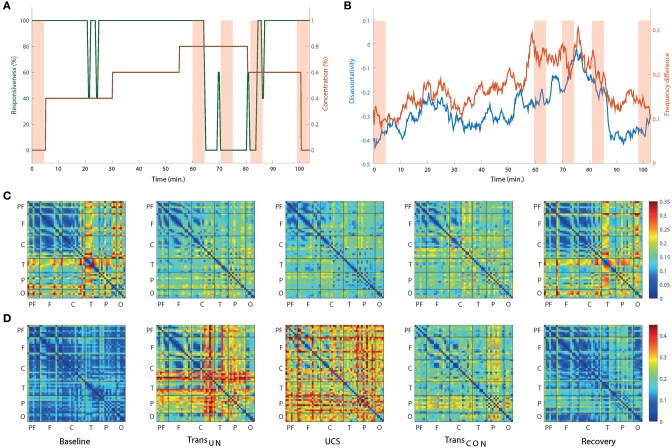
**The network topology and topography of alpha peak frequency associated with ES change across states for one subject as an example**. **(A)** The time evolution of anesthetic concentration (brown line) and responsiveness (green line) over the whole experimental period for a single subject. For the same subject, **(B)** both the disassortativity (blue line) and frequency difference (red line) are variable over time, showing a correlation with the behavioral profiles. **(C)** The average difference of coupling strength and **(D)** the average difference of peak frequency among all nodes are presented to show the overall spatial distributions. The selected five EEG epochs for each subject were averaged over seven subjects. In **(C)**, the large difference of connection strength among specific EEG channels in baseline are reduced such that all connection strengths become more homogenous in UCS. They are restored in recovery. On the contrary, **(D)** shows that the relatively small difference of peak frequency among EEG channels is significantly increased in UCS. The overall changes of connection strength and frequency difference are significant across states. For this display, the EEG channels are grouped in six brain regions (P, prefrontal; F, frontal; C, central; T, temporal; P, parietal; and O, occipital).

Two network properties, disassortativity, and frequency difference, were investigated. Figure 4A presents the timeline of the anesthetic concentration (brown line) and responsiveness (green line) over the experimental period for a single volunteer as an example. Figure 4B presents the disassortativity and frequency difference in coupled nodes for the same volunteer. In the baseline and recovery states, the network showed disassortativity, i.e., a tendency for highly-connected nodes to link with less-connected nodes. However, the overall frequency difference was relatively small with similar peak frequencies among the EEG channels, which could facilitate global synchronization. After induction of unconsciousness, the disassortativity and frequency difference decreased and increased, respectively. Finally, during UCS, the disassortativity was neutralized and the frequency difference reached a maximum. The heterogeneous network topology was changed to a homogenous network topology. Concurrently, the homogeneous frequency distribution changed to a more heterogeneous frequency distribution, making global synchronization more difficult. It is also notable that the disassortativity and frequency difference were closely correlated with even a small change of responsiveness (see around 25 min in Figures 4A,B). The changes of connection strength difference and peak frequency difference among nodes across states are presented in Figures 4C,D, respectively. The results for all individual subjects are presented in Figure S7.

### Relationship of ES conditions with state, anesthetic concentration, and responsiveness

**Figure 5:**
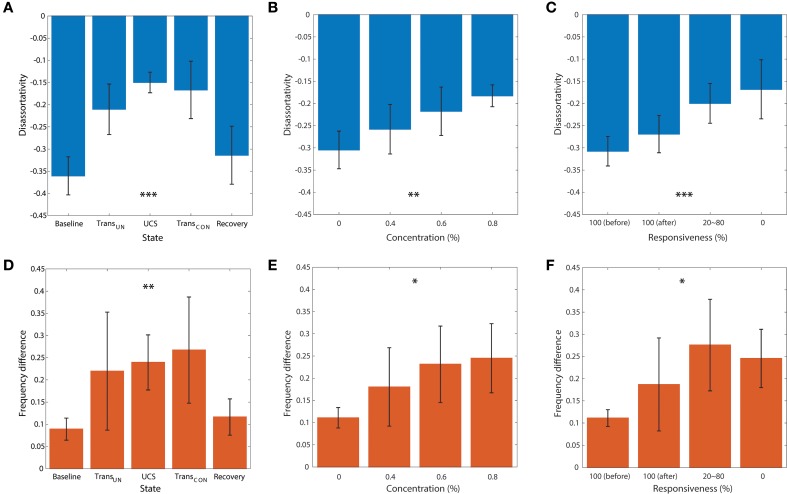
**The relationship of disassortativity and frequency difference to state, anesthetic concentration and responsiveness**. The disassortativity has strong correlations with **(A)** states (baseline, transition to unconsciousness (Trans_*UN*_), unconscious state (UCS), transition to recovery (Trans_*CON*_), and recovery), **(B)** anesthetic concentration (0, 0.4, 0.6, and 0.8%), and **(C)** responsiveness (100% (before), 100% (after), 20–80% and 0%). The frequency difference also showed significant correlations with **(D)** states, **(E)** anesthetic concentration, and **(F)** responsiveness. Disassortativity is reduced along with increased anesthetic concentration and decreased responsiveness. On the other hand, the frequency difference increases along with increased anesthetic concentration and decreased responsiveness. The responsiveness of 100% was separated into two states before induction of unconsciousness and after recovery of consciousness (before and after). Error bar denotes standard deviation. Significance level using ANOVA: *p* < 0.05 “^*^”; *p* < 0.01 “^**^”; *p* < 0.001 “^***^.”

Figures 5A–F demonstrates the average disassortativity and frequency difference over all participants with respect to the states, anesthetic concentration, and responsiveness. The disassortativity and frequency difference were calculated with 30 EEG epochs (10 s long for each epoch) for a participant and then averaged over six participants. We excluded one participant who had only two levels of responsiveness (100 and 0%) in this calculation.

In Figure 5A, the disassortativity was significantly reduced in transitional states and UCS (both, *p* < 0.001), and returned to the baseline level with recovery of consciousness. In contrast, in Figure 5D the frequency difference in coupled nodes significantly increased after induction (*p* < 0.001).

Figures 5B,C,E,F demonstrate the correlations of average disassortativity and frequency difference with anesthetic concentration (0, 0.4, 0.6, and 0.8 %) and behavioral responsiveness (100%[before], 100%[after], 20~80%, 0%), respectively. We divided 100% responsiveness into two behavioral states, before induction and after induction. The disassortativity linearly decreased along with anesthetic concentration (*p* < 0.01) and behavioral responsiveness (*p* < 0.001). Conversely, the frequency difference in coupled nodes significantly increased along with increasing concentration (*p* < 0.05) and decreased responsiveness (*p* < 0.05). Interestingly, even though the participants showed 100% responsiveness before and after sevoflurane exposure, the assortativity, and frequency difference after induction is altered. Thus, the two network properties suggested as ES conditions previously observed in generic networks were significantly enhanced in the brain network during higher anesthetic concentrations, lower responsivity, and unconsciousness.

### Regional and temporal variability of suppression strength for ES

**Figure 6:**
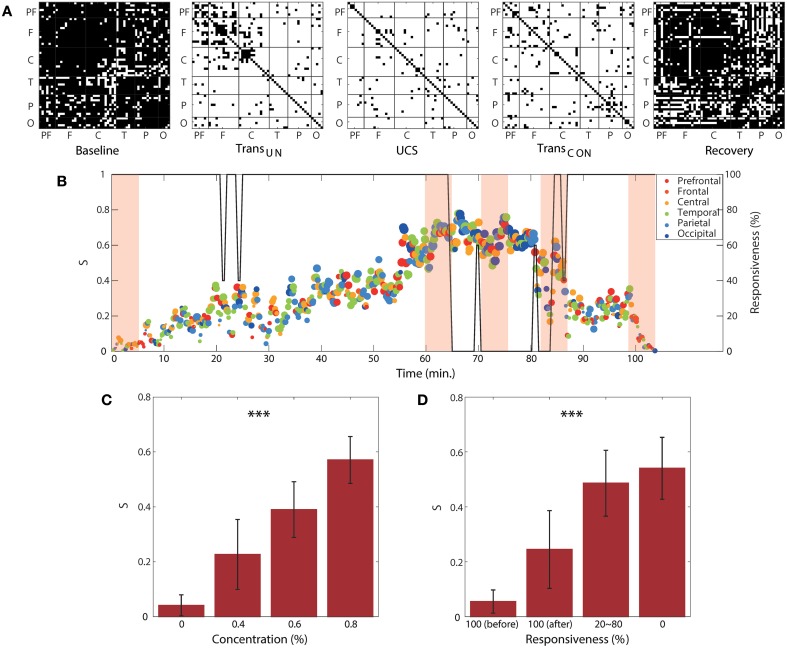
**Regional and temporal variability of suppression strength**. **(A)** Channel pairs satisfying the local suppressive rule for a subject as an example. The pairs of EEG channels that satisfy the local suppressive rule are denoted with white dots (suppressed), whereas the pairs not satisfying the suppressive rule are denoted with black dots (synchronizable). Most pairs of EEG channels satisfy the local suppressive rule after induction, indicating that the synchronization was strongly suppressed in the anesthetized state. For the same subject, **(B)** the global suppression strength S with the brain region that has largest regional suppression strength at each time window is presented over the experimental period. The color and size of circle indicates the brain region and the strength of the largest *S*_*r*_, respectively. The graph shows a large regional and temporal variability of local suppression strength. The shaded areas correspond to the five epochs from which the data in **(A)** were obtained. The size and color of each circle indicate the local suppression strength and the six regions (PF, prefrontal; F, frontal; C, central; T, temporal; P, parietal; and O, occipital), respectively. The responsiveness is indicated by the black line. The average local suppression strength across all subjects showed a strong correlation with **(C)** anesthetic concentration and **(D)** unresponsiveness. Significance level using ANOVA: ^***^p < 0.001.

We observed clear correlations of the two global network properties (disassortativity and frequency difference) with anesthetic concentration and responsiveness. We examined this relationship with local ES conditions. Figure 6A demonstrates the pairs of EEG channels that satisfy the local suppressive rule across the five states for a single volunteer. In the baseline and recovery states, most pairs of EEG channels did not satisfy the suppressive rule (denoted with black dots in Figure 6A), implying that they may contribute to gradual synchronization path rather than ES. However, in the two transitional states and unconscious state, the most highly coupled nodes in the brain network satisfied the suppressive rule with significantly increased frequency difference in the coupled nodes and a reduced threshold. This threshold is determined by the product of decreased coupling strength and phase synchronization for each state (denoted with white dots in Figure 6A). In this condition, the coupled nodes are more likely to provoke ES.

The regional suppression strength, *S*_*r*_, was defined by the proportion of connected nodes that satisfy the local suppressive rule in a region. The regional suppression strength has a value, 0 ≤ *S*_*r*_ ≤ 1. If *S*_*r*_ = 1, it indicates that all connected nodes satisfy the rule and the regional network facilitates an ES. Otherwise, if *S*_*r*_ = 0, all connected nodes violate their local rules, thus, it is highly probable for the region to follow a gradual synchronization path. Figure 6B shows the temporal and regional variability of the suppression strength. The brain region with the largest *S*_*r*_ among the six regions was presented at each 10 s interval. Figure 6C demonstrates the significant change of the average global suppression strength over all subjects with an increase of anesthetic concentration (*p* < 0.001) and decrease of responsiveness (*p* < 0.001). In particular, the average suppression strength *S* for the anesthetic concentration of 0.8% (0.57 ± 0.09) was approximately 10-fold higher than that of 0% concentration (0.04 ± 0.04). The average suppression strength *S* for 100% responsiveness (0.05 ± 0.04) was also about 10-fold higher than that of 0% responsiveness (0.54 ± 0.11) in Figure 6D. Note that in comparison with two network properties that have about 3-fold differences in Figure 5, the average *S* is more sensitive to the change of anesthetic concentration and responsiveness. Importantly, it quantitatively indicates that at the point where 50% of connected node pairs in the brain network are suppressed, responsiveness is lost. Furthermore, *S* clearly differentiated the two states of 100% responsiveness (before and after induction, *p* < 0.05), showing that the number of suppressed node pairs was significantly increased from about 5 to 20% in the 100% responsive state after recovery from anesthesia (100%[before] vs. 20 ~ 80%: *p* < 0.001; 100%[before] vs. 0%: *p* < 0.001; 100%[after] vs. 20 ~ 80%: *p* < 0.05; 100%[after] vs. 0%: *p* < 0.001; 20 ~ 80% vs. 0%: *p* < 0.05), which indicates a relatively higher possibility for a sudden state transition back to unconsciousness. The results for all individual subjects are presented in Figure S8.

### Two representations of gradual and abrupt transitions with suppression strength and synchronization

**Figure 7:**
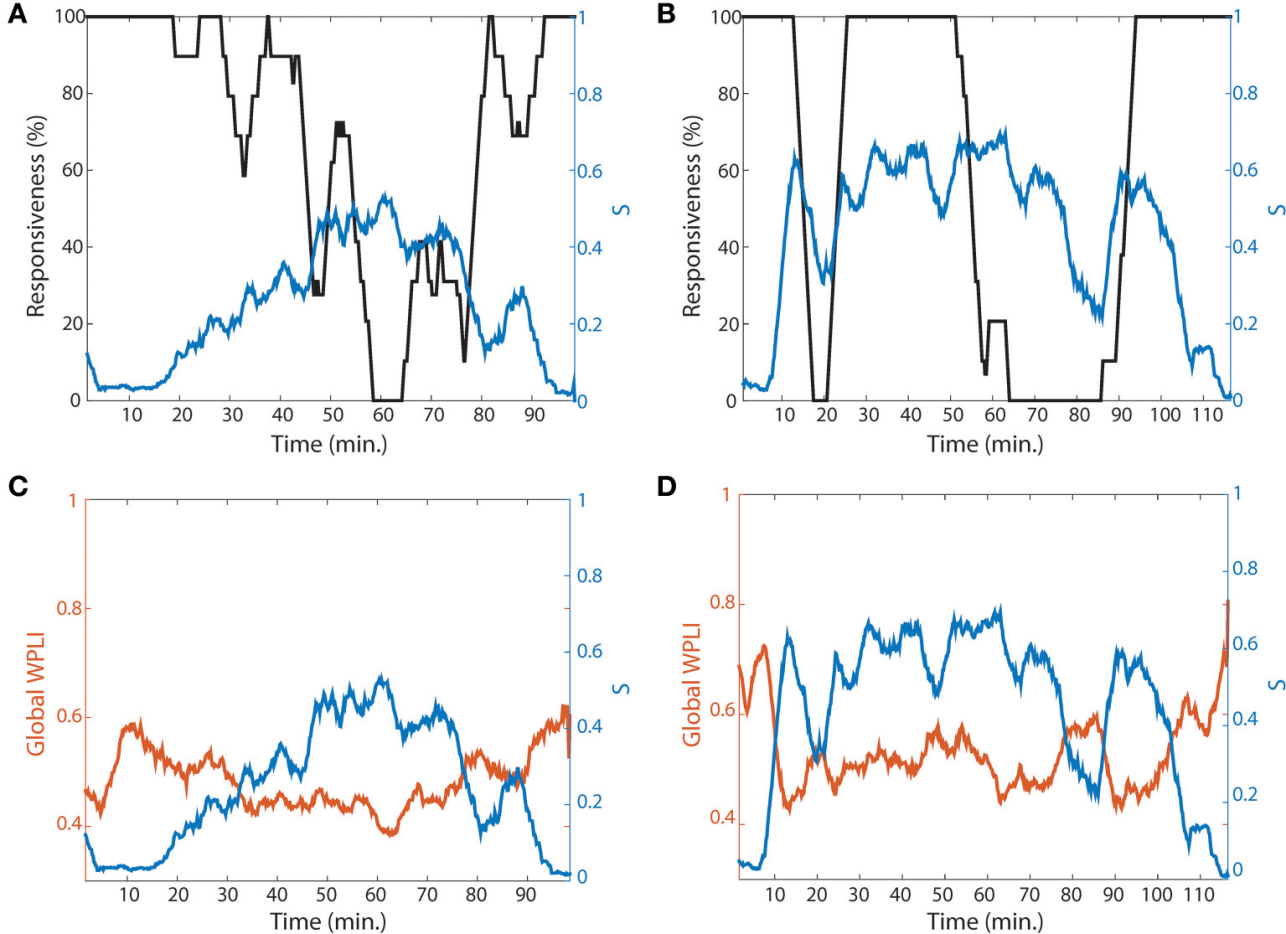
**Two representative subjects for gradual and abrupt state transitions**. The responsiveness (black line) and suppression strength *S* (blue line) of **(A)** the gradual (subject #4) and **(B)** abrupt state transition (subject #10) are presented. The responsiveness was averaged by 5 min time period with shifting 30 s to show the trend of temporal change. In comparing the two subjects, the gradual state transition has a lower *S*, whereas the abrupt state transition has a relatively higher *S*. **(C,D)** present the global *WPLI*s (orange line), averaged over all connected pairs of EEG channels for each subject, to see the association of suppression strength with the change of network state (synchronization) as well as behavioral state (responsiveness). The global *WPLI* in the gradual state transition showed a smaller change, while the abrupt state transition showed precipitous changes during the experimental period.

Even though the four network conditions indicate a higher possibility of ES during the lightly anesthetized state, the design of current study was limited because we did not attempt to arouse the volunteers to assess whether ES indeed occurs when the conditions are met and a network perturbation occurs. However, as one method to support our hypothesis, we present two volunteers whose recovery trajectories represent gradual and abrupt state transitions to responsiveness. One volunteer in Figure 7A shows slow loss and recovery of responsiveness. In order to show a trend of temporal change, the responsiveness was smoothed by averaging 5 min long time window and moving it 30 s. It took about 40 min for the subject in Figure 7A to reach the point of 0% responsiveness (from 20 to 60 min in Figure 7A, black line), and 25 min to recover back to 100% responsiveness (from 65 to 90 min). In contrast, the other subject in Figure 7B showed fast loss and recovery of responsiveness. The participant first lost responsiveness around 10 min in Figure 7B; the time taken for loss and recovery of 100% responsiveness were about 5 min. Correlating this with a change of network properties, the two subjects demonstrated dramatically distinct patterns of suppression strength S and *global WPLI* (Figures 7C,D). Here we considered the *global WPLI* between connected node pairs as a measure of reliable phase synchronization in EEG data. For the subject who showed a gradual state transition, the suppression strength *S* also increased gradually with a relatively low overall *S*, and the change of synchronization was small and not much different from that of baseline. The corresponding synchronization of the brain network was also relatively small and demonstrated gradual changes. On the contrary, the subject who showed an abrupt state transition had higher suppression strength *S* before the first large drop of responsiveness (around 10 min in Figure 7B), maintaining large *S* during the anesthetized state and with another episode of fast loss and recovery of responsiveness at 50 min. For this subject, the synchronization in baseline was precipitously decreased along with a steep decrease of *S*. The other subjects showed combined patterns of gradual and abrupt transitions. The results for all individual subjects are presented in the Figure S9.

## Discussion

General anesthesia is administered clinically tens of millions a time each year and is also a powerful tool to modulate the transition between conscious and unconscious states. During anesthesia, the ability of the brain to process information is disrupted with characteristic alterations of its functional network structure and dynamics (Alkire et al., 2008; Boveroux et al., 2010; Ferrarelli et al., 2010; Ku et al., 2011; Monti et al., 2013; Lee et al., 2013a,b; Barttfeld et al., 2015). How brain networks are able to rapidly recover to normal conscious functioning after the discontinuation of the anesthetic has not been elucidated. This clinically and neuroscientifically important question has broad implications for the assessment and potential recovery of consciousness in pathological states of unresponsiveness and coma.

Anesthetic state transitions associated with the loss and recovery of consciousness have been investigated with various approaches, including mathematical models, genetic manipulation, advanced signal processing, and graph-theoretical network analysis (Steyn-Ross et al., 1999, 2001a,b, 2003, 2004; Friedman et al., 2010; Lee et al., 2011; Purdon et al., 2013; Chander et al., 2014; Hudson et al., 2014). Steyn-Ross et al. (1999) developed the first model for the anesthetic effect on neural activity at the macro-column scale, which is an assembly of inhibitory and excitatory neurons in a volume of diameter of about 1 mm and depth of 3 mm, containing about 100,000 neurons. In this model study, they reported that a critical anesthetic concentration (the primarily GABAergic anesthetics propofol and sevoflurane) causes a sudden state transition, reminiscent of a first-order phase transition of thermodynamics, which is characterized by a hysteresis between the induction of and emergence from anesthesia. Recently, Hudson et al. (2014) used advanced signal processing of electrophysiological data from rats to show that when the anesthetic is discontinued, the brain recovers through an ordered series of state transitions and distinct spatiotemporal activity patterns. Some transition paths are highly probable, whereas others are less probable. Lee et al. (2011) applied a novel graph-theoretic network analysis to human EEG during and after anesthesia, dissociating the effects of network structure and connection strength on global efficiency across states. They identified two types of state transitions at loss and recovery of consciousness: “slow decay and sudden return” (8 out of 20 subjects) and “sudden decay and slow return” (12 out of 20 subjects), suggesting elements of both discrete and continuous state transitions possible. Purdon et al. (2013) focused on the characteristics of EEG during state transition induced by propofol. They noted an increase in median frequency of the alpha band during emergence, and observed distinct patterns of cross-frequency coupling between the phase of slow-wave oscillations (0.1–1 Hz) and the amplitude of the alpha (8–13 Hz) band in deep anesthesia and during the return of consciousness. Blain-Moraes et al. suggested that the typical pattern of amplitude-phase coupling is drug-specific rather than state-specific, by comparing the results of propofol to those of ketamine and sevoflurane (Blain-Moraes et al., 2014, 2015). Hight et al. (2014) used a Bayesian method to estimate the likelihood of EEG patterns that map the patient's state to 2-dimensional manifolds in a state space of excitatory connection strength vs. the change in intrinsic resting neuronal membrane conductivity. They observed two types of state transition in the state space: archetypal emergence showed a progressive decrease in alpha power and increase peak alpha frequency before return of responsiveness, whereas non-archetypal emergence demonstrated no spectral EEG change and an abrupt return of responsiveness. A similar study was also carried out by Chander et al. (2014) classifying the emergence patterns of 100 surgical patients into two types of emergence (progressive and abrupt) based on the power spectrums of delta (0.5–4 Hz) and alpha/spindle(8–14 Hz) of frontal EEG. Friedman et al. (2010) and Joiner et al. (2013) suggested that the central nervous system has a tendency to resist behavioral transitions between the conscious and unconscious states, which they term “neural inertia.” Neural inertia has been proposed to account for the hysteresis between entering into and emerging from the anesthetized state. Furthermore, they demonstrated that genetic mutations affecting sleep-wake circuitry modulate the hysteresis with multiple anesthetics in both flies and mice.

The current study investigated brain network conditions including network topology, local dynamics, and a threshold for ES determined by the interaction of global topology and local dynamics. The significance of these properties in predicting the relative probability of the two types of state transitions has recently been studied in generic, non-biological networks. Using quantitative and empirical evidence, this study demonstrates, for the first time, that the network conditions for ES are also present in brain networks during pharmacological perturbation. This suggests that mechanistic explanations for abrupt and gradual state transitions in general complex networks might also be applicable to the brain.

We found that light sevoflurane anesthesia reconfigured network topology (BC, GE, modularity, and disassortativity) and dynamics (peak frequencies and differences between them in coupled nodes), as well as the correlation between network topology and dynamics (node degree vs. peak frequency) in the transitional and unconscious states. We observed that the high degree of disassortativity and strong hub structures in the resting state of baseline consciousness is diminished significantly after induction of anesthesia. Our interpretation is that the disruption of hub structures after induction prevents the hub regions from functioning as “seeds” of gradual synchronization. At the same time, the frequency difference of coupled nodes gradually increased after induction and reached a maximal value in the unconscious state. The increase in frequency difference of coupled nodes hinders their synchronization and thus also delays global synchronization. Regarding the correlation between node degree and frequency, a salient positive correlation appeared in the unconscious state, whereas no correlation was present in baseline and recovery states. After induction, the broad range of node degree contracted to a homogeneous network with disrupted hubs and the peak frequency of alpha band increased from about 9.5 to 11.0 Hz.

Surprisingly, the broadly distributed peak frequencies were positively correlated with the node degrees in the unconscious state. This frequency-degree relationship in complex networks was first introduced as a condition for ES and opened active discussions in the network science community, followed by discoveries of other such conditions. Furthermore, applying the modified suppressive rule to the unconscious state, the increased frequency difference of coupled nodes deviated from the significantly reduced threshold (i.e., the product of reduced coupling strength and reduced phase synchronization). Since the suppressive rule was derived as a necessary condition for synchronization in coupled nodes, the pervasive violations of this rule indicated that synchronization is strongly suppressed in the unconscious state. In practice, the suppression strength outperformed the conventional network properties in the differentiation of states (10-fold difference vs. 3-fold difference), suggesting it as a possible index for level of consciousness. As a result, all four network conditions for ES investigated here provide support for the hypothesis that the anesthetic sevoflurane suppresses global synchronization by reconfiguring the network topology and dynamics in the brain. The inability to achieve temporal coordination across the network is consistent with the observed increase in modularity during unconsciousness.

The four network conditions (increased frequency difference, reduced disassortativity, positive correlation between node degree and frequency, and pervasively held suppressive rule) and their correlations with anesthetic concentration and responsiveness suggest that brain networks just past the loss of consciousness are primed for recovery. Consistent with clinical experience, a small perturbation—such as enhanced connectivity from an external stimulus or small change of anesthetic concentration—could give rise to an abrupt state transition back to the normal network. Further work is required to assess the conditions for ES during deeper anesthetic states (as achieved during surgery). Assessing the conditions for ES in pathological states such as coma or unresponsive wakefulness syndrome might provide prognostic insight into the likelihood of recovery. Finally, the physiological state of sleep-induced unconsciousness must also be investigated, since this state would potentially have an even higher propensity to ES than the light state of anesthesia analyzed in the current investigation.

### Limitations

This investigation has a number of limitations. First, we only studied a lightly anesthetized state using relatively low doses of sevoflurane. Further study on deep anesthesia is needed to better understand this phenomenon and its relevance to states of consciousness. Second, there is a possibility that different anesthetic drugs could induce different types of transition. Therefore, drug-specific effects on the ES conditions need to be elucidated. Third, we assumed that the coupling strength of the brain network was inversely related to the anesthetic concentration, but the actual relationship in large-scale brain networks must be determined empirically in further studies. Fourth, it is still not clear why the alpha band of the EEG was the only one that demonstrated significant network changes across the states. This could reflect the relationship of alpha rhythms to cognitive functions. Furthermore, because the alpha rhythm is the only consistent narrow-band oscillation in the EEG, it may be more amenable to the coupled Kuramoto model compared to other bands and the 1/f characteristic of the broad band that make it difficult to define a representative frequency. Fifth, despite the robustness of *WPLI* against potentially confounding volume conduction, *WPLI* is relatively insensitive to phase differences at high frequency. Therefore, we only performed the analysis within the lower frequency bands (delta, theta, alpha, and beta). Sixth, the question of whether the four conditions are sufficient or necessary and whether or not they come from one condition is beyond the scope of this study. With further study in model networks, one could find the minimal (sufficient or necessary) condition for ES, and its relationship with human brain network connectivity. Seventh, we did not demonstrate a causal relationship between the conditions for ES and the actual occurrence of ES. However, it is known that relatively innocuous stimuli can reverse behavioral state during light anesthesia and we suggest that conditions of ES explain this easy reversibility. The next step will be a demonstration of the causality of the conditions to produce actual ES, perhaps by implementing a form of stochastic perturbation in a large-scale brain network model.

## Conclusion

Sevoflurane disrupts efficient network topology and dynamics of the human brain. At an anesthetic concentration just past the threshold of unconsciousness, we identified the development of four different network conditions for ES in human brain networks that have only been reported in network model studies. This quantitative evidence provides a possible explanation for how disrupted brain networks during anesthesia can rapidly recover as the suppressive force of the anesthetic vanishes. The present investigation explores for the first time the presence of network conditions for ES in an empirically derived human brain network as a potential mechanism of recovery from pharmacologically-induced unconsciousness.

## Author contributions

MK analyzed the data and wrote the paper. SM, GV, VT, and EJ were involved in the data acquisition. GM and AH contributed to writing the paper. UL conceived of the study design and wrote the paper.

### Conflict of interest statement

The authors declare that the research was conducted in the absence of any commercial or financial relationships that could be construed as a potential conflict of interest.
