# Supplementary material for: Functional and Topological Conditions for Explosive Synchronization Develop in Human Brain Networks with the Onset of Anesthetic-Induced Unconsciousness
Source: Front Comput Neurosci. 2016 Jan 21;10:1. doi: 10.3389/fncom.2016.00001 (PMC4720783; doi:10.3389/fncom.2016.00001)
Supplement: Supplementary file 1 [file Presentation1.PDF]

Network properties derived from non-alpha bandwidths

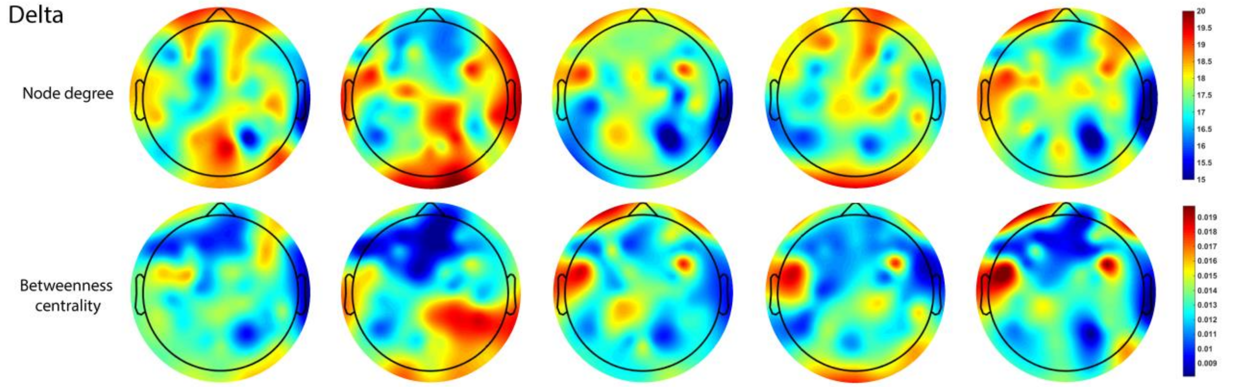

Figure S1. Topography of the average node degree and betweenness centrality in delta band (1 - 4 Hz).

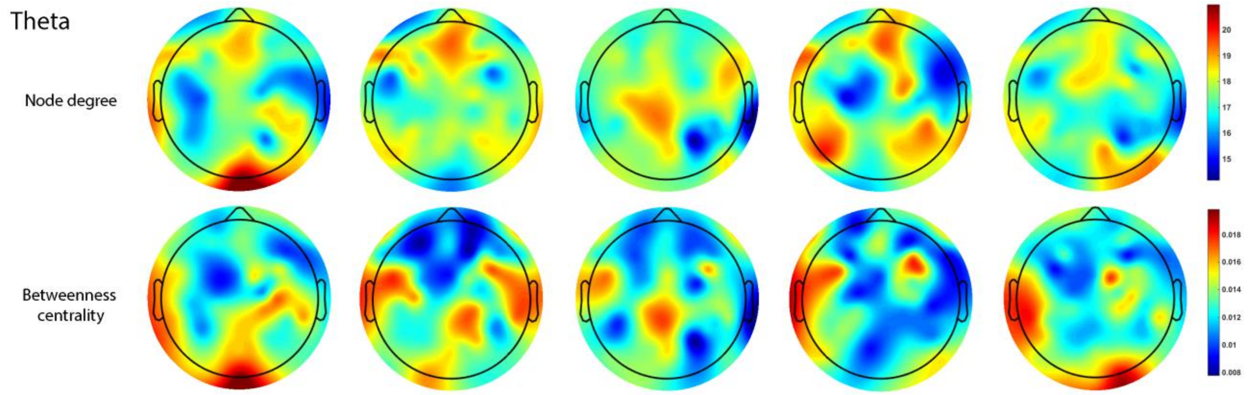

Figure S2. Topography of the average node degree and betweenness centrality in theta band (4 - 8 Hz).

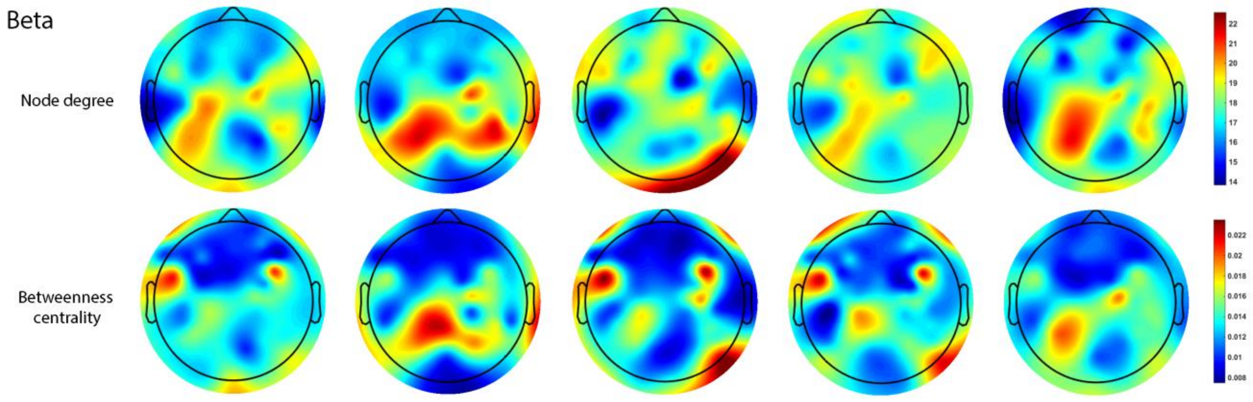

Figure S3. Topography of the average node degree and betweenness centrality in beta band (13 - 25 Hz)

There was no state-specific topographic pattern observed in the comparison of node degree and BC distribution across the five states: baseline, two transitions, unconsciousness state, and recovery state. The node degree and BC were calculated in the same way as alpha.

Peak frequency of broadband EEG (4 – 30 Hz)

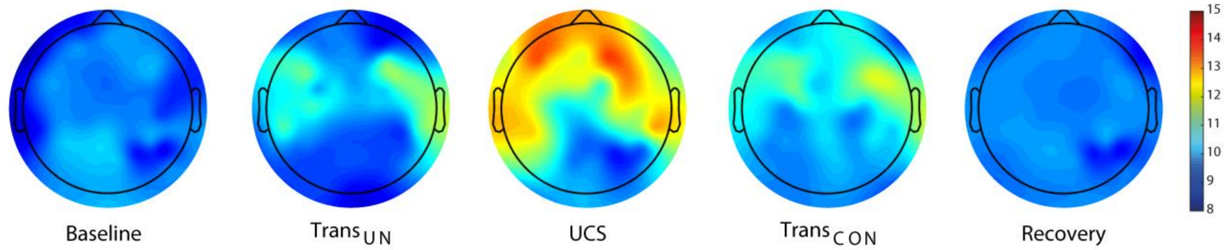

Figure S4. Topography of the average peak frequency within broadband EEG (4 - 30 Hz). All peaks for each channel and each state are within the alpha frequency range.

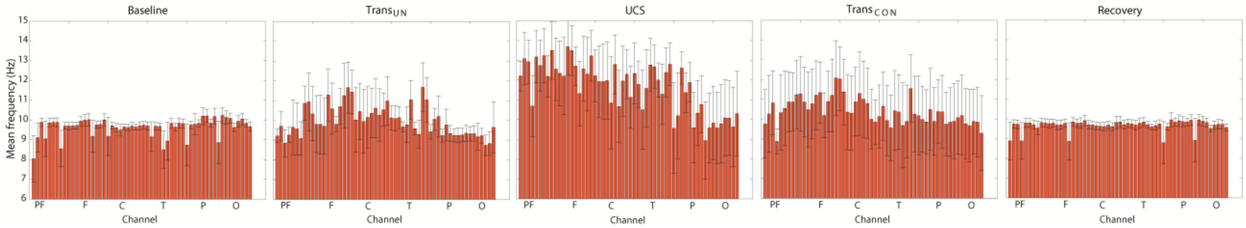

Figure S5. Average peak frequency of each channel for each state within broadband (4 – 30 Hz). EEG channels are aligned in region from prefrontal to occipital (PF: prefrontal, F: frontal, C: central, P: parietal, and O: occipital). Error bar denotes standard error. The peak frequencies of channels are homogenously distributed around 10 Hz. However, increase of peak frequency in anterior regions is observed in unconscious state.

To support the results in and subsequent focus on the alpha band, we investigated where the peak frequency is located within broadband EEG (4-30Hz). We observed every peak is located within alpha frequency range for each channel and for each state. There is a clear shift of peak frequency to the frontal region and an increase of peak frequency during unconsciousness.

Correlation between degree and peak frequency

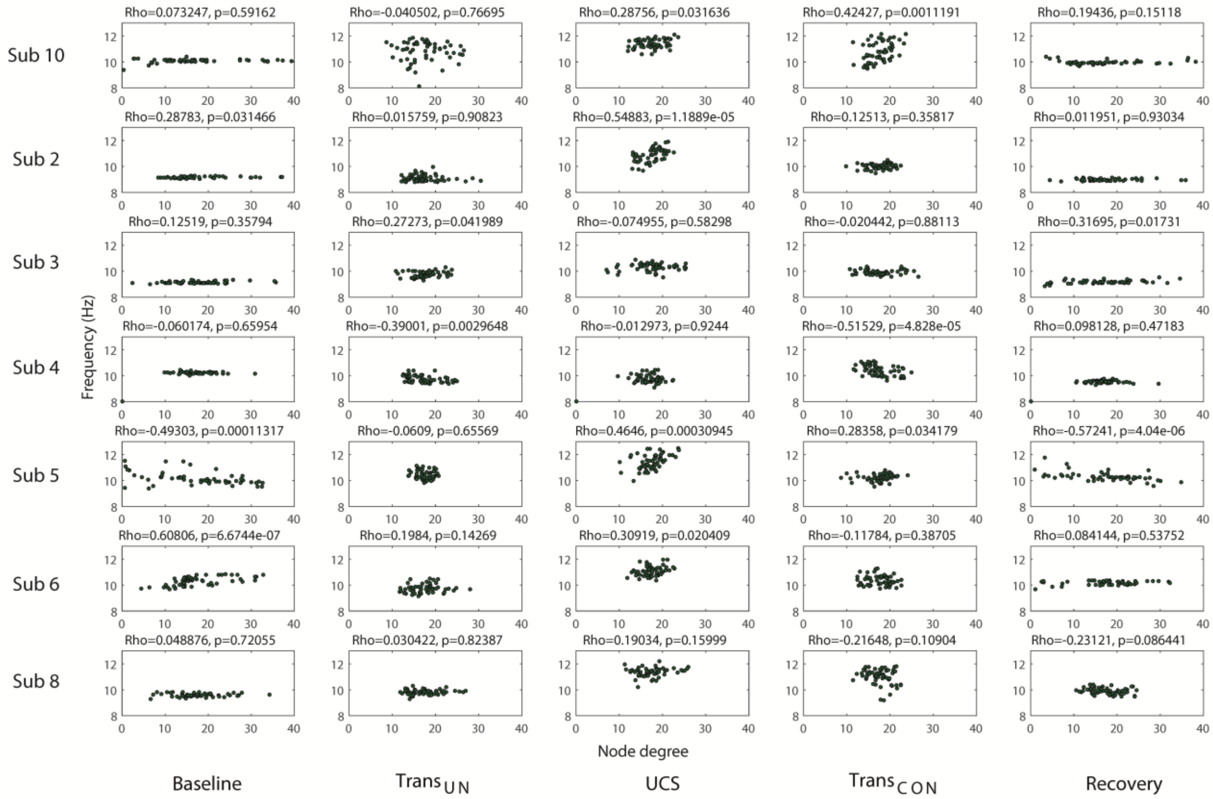

Figure S6. Spearman correlation between node degree and peak frequency for individual subject. The correlation value 'Rho' is presented in each figure and 'p' is a significance of each spearman correlation.

The network topology and dynamics within the alpha band for individual subjects

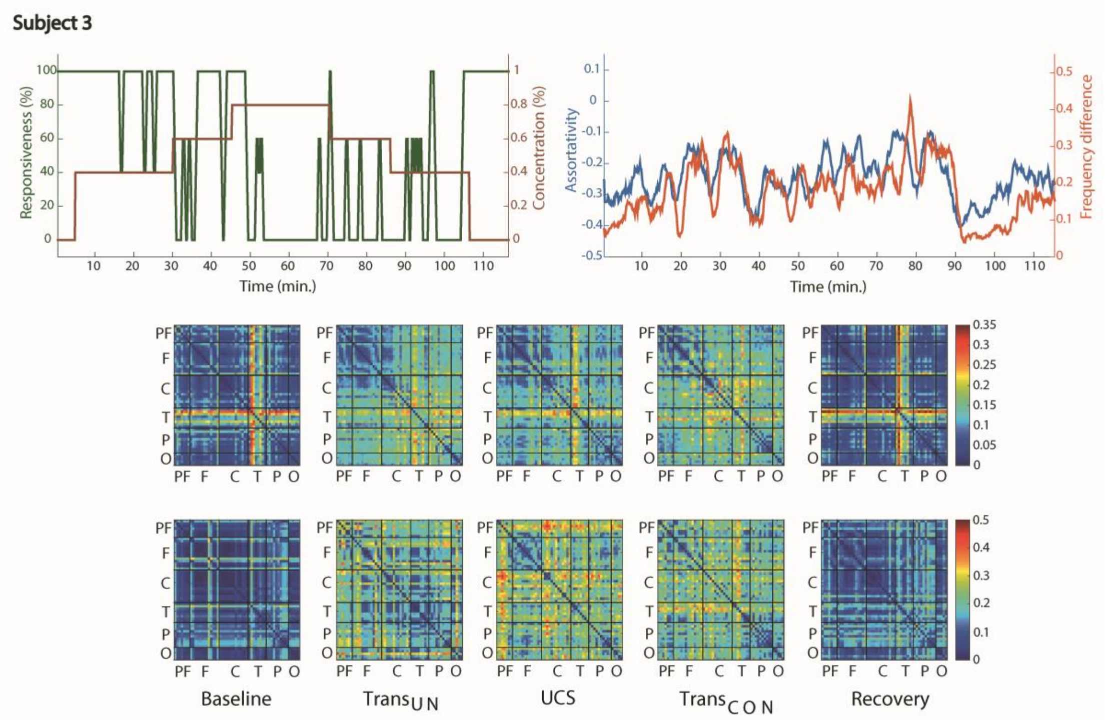

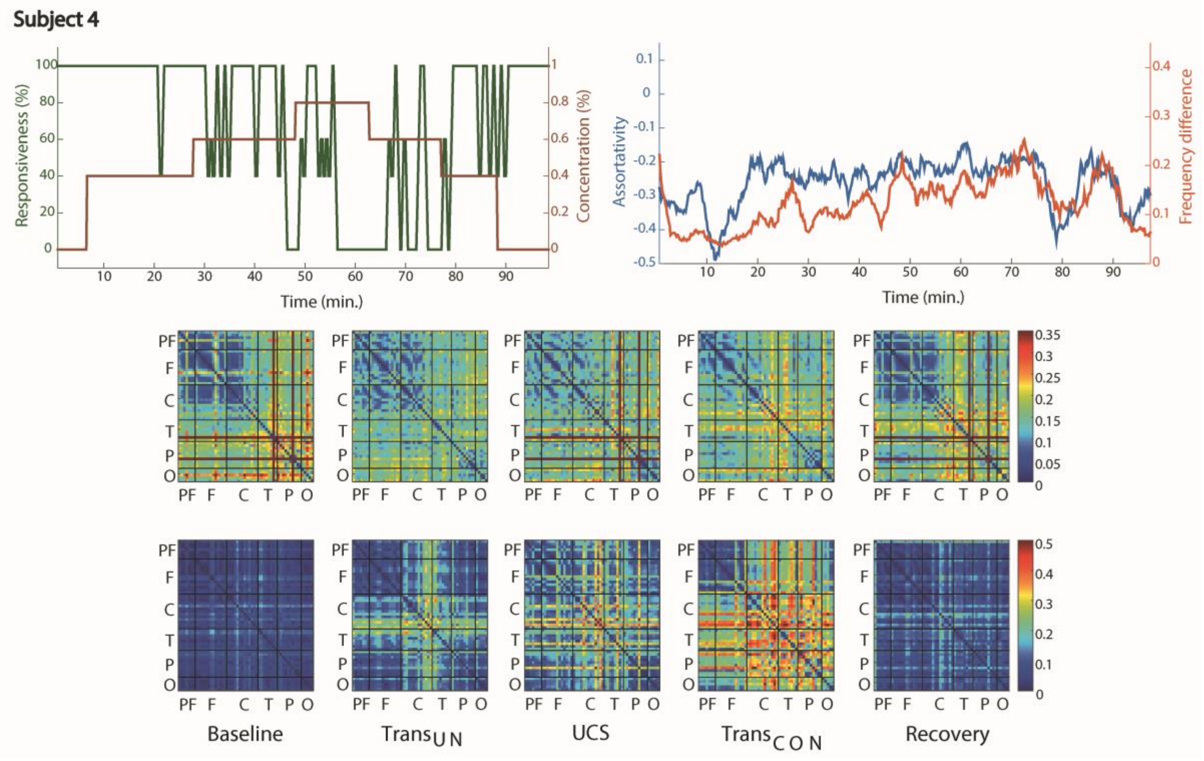

Subject 5

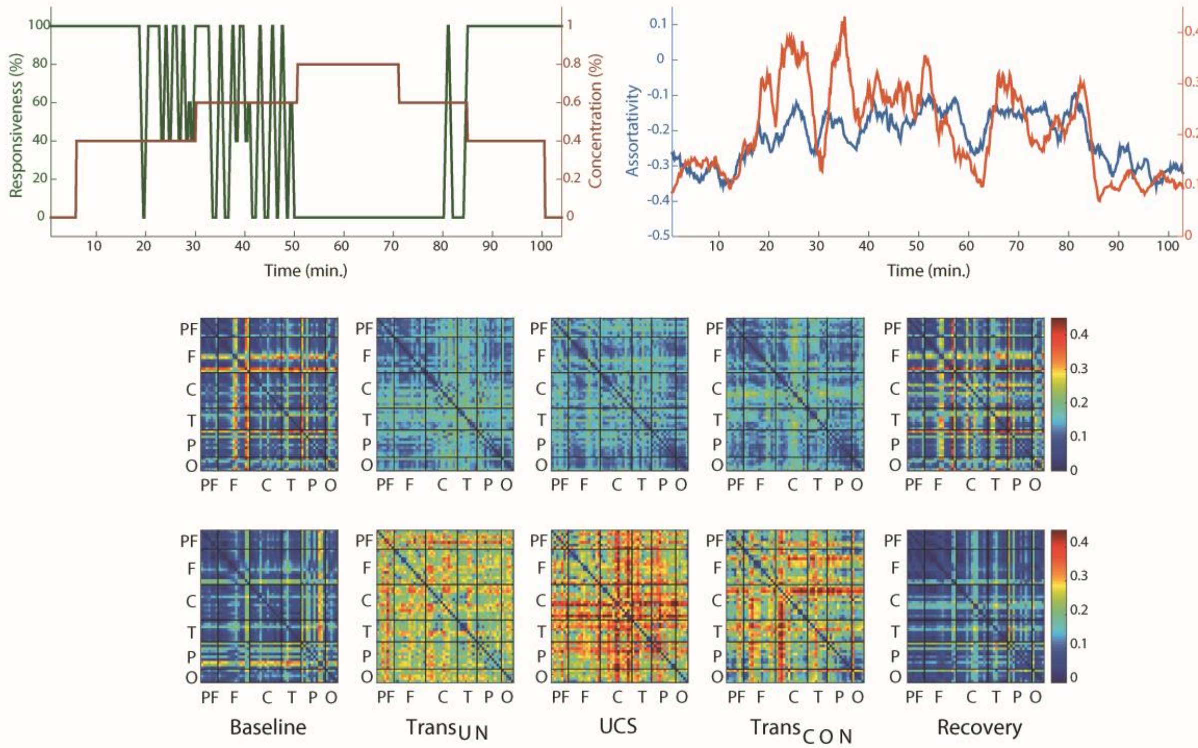

Subject 6

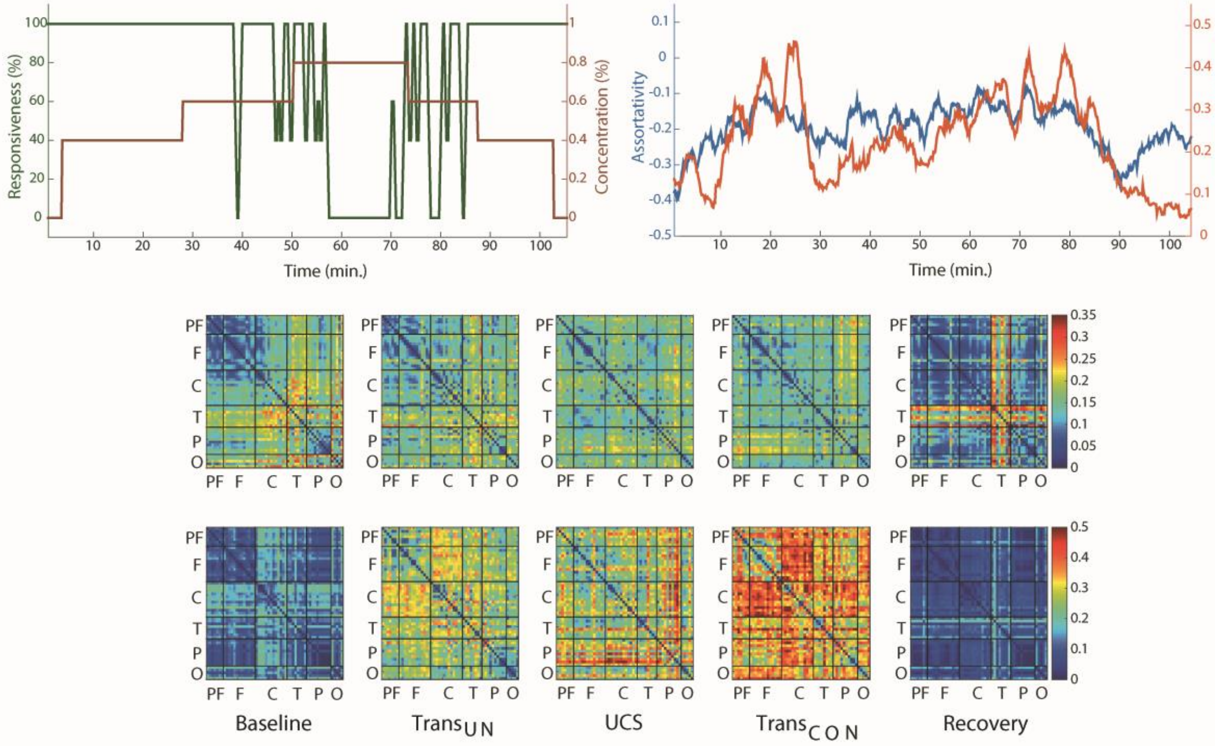

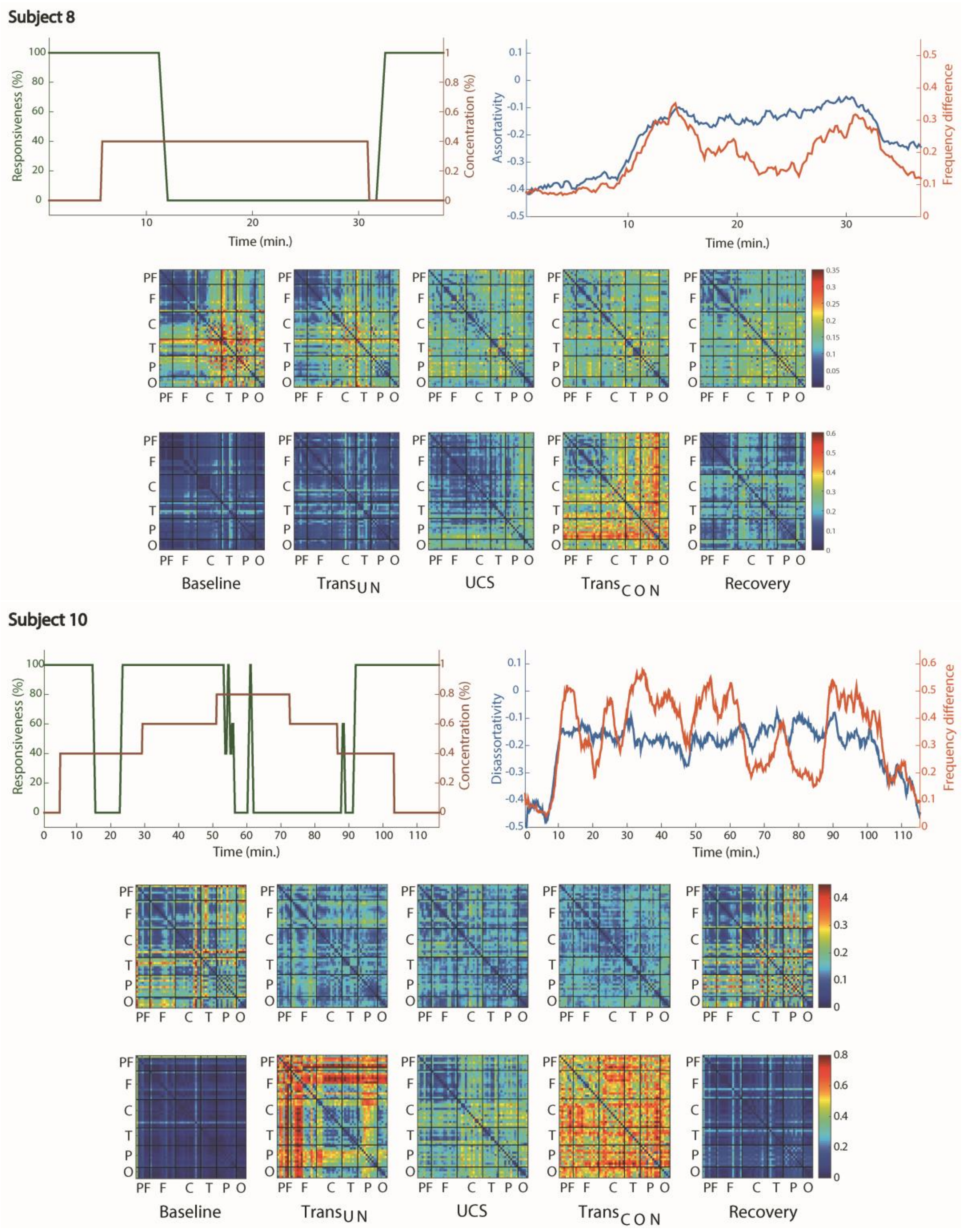

Figure S7. Network topology and dynamics for individual subjects (all denotations are the same as Fig.4 in the original paper).

Regional and temporal variability of the suppression strength for individual subject

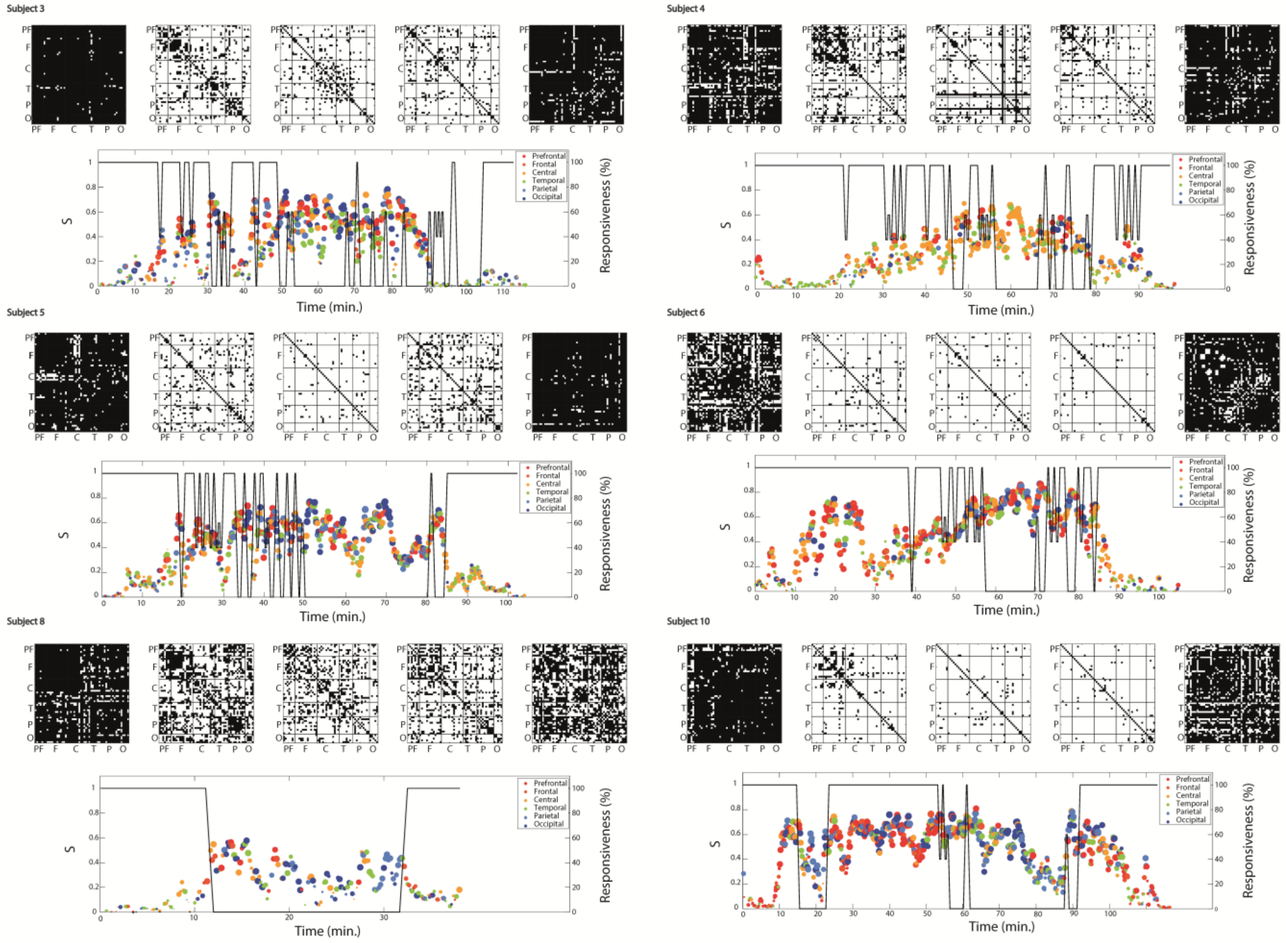

Figure S8. Regional and temporal suppression strength for individual subject (all denotations are the same as Fig.6 in the original paper).

Suppression strength and synchronization with responsiveness for individual subjects

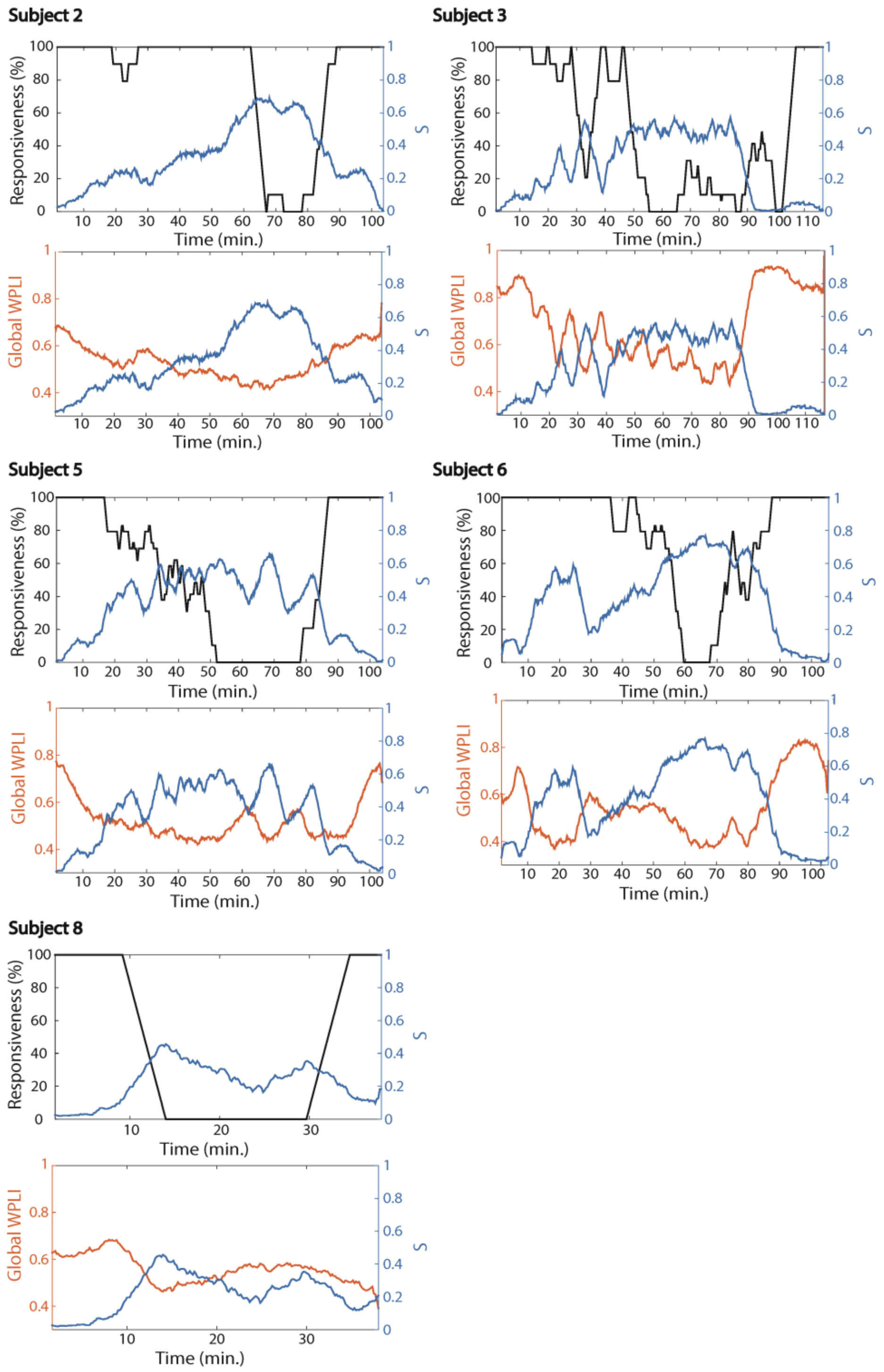

Figure S9. Suppression strength and synchronization with responsiveness (all denotations are the same as Fig.7 in the original paper).
